# Editorial: Volume II: tumour microenvironment in cancer research and drug discovery

**DOI:** 10.3389/fphar.2026.1929346

**Published:** 2026-09-17

**Authors:** Nur Akmarina B. M. Said, Syed Mahmood, Kenneth K. W. To

**Affiliations:** 1 Department of Pharmaceutical Life Sciences, Faculty of Pharmacy, University of Malaya, Kuala Lumpur, Malaysia; 2 Department of Pharmaceutical Technology, Faculty of Pharmacy, University of Malaya, Kuala Lumpur, Malaysia; 3 School of Pharmacy, Faculty of Medicine, The Chinese University of Hong Kong, Hong Kong, Hong Kong SAR, China

**Keywords:** cancer, drug repurposing, immune cells, immunotherapy, tumour microenvironment

From being a passive backdrop, the tumour microenvironment (TME) is now recognised as a core driver of cancer initiation, progression, metastasis, immune escape, and treatment response. As such, there is a major shift from cancer-centric to TME-centric research, positioning TME as a hub for therapeutic targets. Approved and experimental strategies are now directed towards various components and conditions of the TME, including immune checkpoints, cancer-associated fibroblasts, ECM, and inflammatory niches, as well as processes like angiogenesis, metabolism, hypoxia, and acidity (Goenka et al., 2023) aiming to advance precision oncology, inform prognostic biomarkers (e.g., Immunoscore), and optimize combination regimens in cancer management (Tonello et al., 2025).

The intricate and interdependent network of tumour microenvironment (TME) components, together with tumour heterogeneity, continues to establish key players within this complex ecosystem. In this Research Topic, we highlight recent advances in overcoming the biological, immunological, and physical barriers of the TME to enhance the efficacy of cancer treatments, particularly immunotherapy. These span elegantly around pharmacology, immunology, and collectively outline a unified, multimodal attack on cancer.

One of the central themes emerging from this Research Topic is the exploitation of specific biochemical and metabolic pathways within the TME that enable tumours to evade immune surveillance. This include the inhibition of TIGIT, a CD-155 ligand binding receptor on T cells and Natural Killer (NK) cells that is responsible for immune cell exhaustion. Tiragolumab targets this pathway by blocking TIGIT signalling, thereby restoring effector cell function and revitalizing anti-tumour immunity (Ghasemi et al.). Similarly, tumour-driven metabolic reprogramming involving indoleamine 2,3-dioxygenase (IDO) and tryptophan 2,3-dioxygenase (TDO) depletes essential amino acids, metabolically starving effector T cells, promoting T-cell apoptosis, and driving the expansion of highly immunosuppressive Regulatory T cells (Tregs) and Myeloid-Derived Suppressor Cells (MDSCs) (Al-Zoubi et al.).

Beyond metabolic reprogramming, the immunosuppressive landscape of the TME is further reinforced by chronic inflammatory signalling pathways. Among these, the COX-2/PGE2 axis plays a pivotal role in sustaining a pro-tumour inflammatory milieu by recruiting Tregs and MDSCs while suppressing the activity of NK cells and CD8^+^ T cells. Targeting this pathway with the selective COX-2 inhibitor celecoxib effectively reduces PGE2 production, thereby disrupting this immunosuppressive circuitry and restoring a more favourable environment for anti-tumour immune responses (Kuang et al.).

Another prominent theme is overcoming the challenge of ‘cold’ or immune-excluded tumours, which physically restrict or entirely lack T-cell infiltration and are therefore notoriously resistant to conventional immunotherapies. In this issue, strategies aimed at igniting anti-tumour immune responses are explored from the perspectives of immunogenic cell death (ICD) and CD4^+^ T-cell modulation (Zhao and Dai; Shahreen et al.). Harnessing targeted therapies, radiotherapy, or chemotherapy can induce a specific form of regulated tumour cell death that triggers ICD, leading to the release of Damage-Associated Molecular Patterns (DAMPs) such as ATP, calreticulin, and HMGB1. These distress signals recruit and mature dendritic cells (DCs), leading to a massive influx of cytotoxic T lymphocytes that turn the tumour ‘hot’.

Recent approaches have also shifted towards the selective modulation of CD4^+^ T cells, moving beyond the traditional view of these cells as merely passive helpers. Specific CD4^+^ T-cell subsets secrete cytokines, including IFN-γ, that play critical roles in orchestrating cytotoxic immune responses and recruiting NK cells. As such, targeted modulation of these populations represents a promising strategy to reprogramme an immune-tolerant TME into a hostile environment for tumour growth and survival.

Beyond the manipulation of targetable molecule pathways, engineering therapeutics to overcome the physical and fluid-dynamic barriers of the TME remains a significant challenge. Therapeutic delivery to solid tumors is often hindered by elevated interstitial fluid pressure and a dense stroma architecture, both of which severely restrict conventional pressure-driven drug perfusion. In this issue, a computational reverse advection-diffusion (RAD) model reveals that exploiting electrohydrodynamic (EHD) forces generated by the electric charge of the glycocalyx at the walls of tumor blood vessels can actively drive plasma and therapeutic solutes across these physical barriers, thereby enhancing perfusion into the tumor matrix (Akash et al.).

The integration of nanotechnology with immunological insights for immunotherapy delivery is also explored, particularly through nanoscale delivery systems such as liposomes, dendrimers, and polymeric nanoparticles. These platforms protect therapeutic payloads from enzymatic degradation while enabling targeted delivery to immune cells, including CD4^+^ T cells. As a result, they minimize off-target systemic toxicity and facilitate more effective penetration of therapeutics into the TME.

Collectively, the studies featured in this issue underscore the growing shift towards synergistic and multimodal therapeutic strategies to address the limitations of monotherapies in overcoming tumour heterogeneity, compensatory escape mechanisms, and the complex resistance landscape of the TME. This is exemplified by the rational combination strategies across diverse therapeutic platforms.

TIGIT Blockade, for example, demonstrates synergistic activity when combined with PD-1/PD-L1 checkpoint inhibitors (like atezolizumab), thereby preventing tumours from relying on alternative immune checkpoints pathways.

Likewise, IDO/TDO Inhibitors are being actively investigated in combination with cancer vaccines and immune checkpoint inhibitors (ICIs) (Al-Zoubi et al.). Celecoxib serves as a versatile sensitizing agent that augment the efficacy of standard chemotherapy, radiotherapy, and immunotherapy by mitigating inflammation-driven resistance mechanisms (Kuang et al.). Similarly, both ICD-inducing therapies and nanomedicine-based delivery platforms are explicitly designed to complement ICIs to maximize and prolong anti-tumour immune responses (Zhao and Dai; Shahreen et al.).

Together, these advances highlight that the strategic integration of complementary approaches targeting the biological, immunological, metabolic, and physical barriers of the TME is essential. While rational combination therapies represent the future direction of TME-centric interventions, the development of more representative models, including 3D cultures, organ-on-chip systems, and microfluidic platforms (Tiwari et al., 2022) - remains critical for accurately predicting therapeutic responses and enabling deeper investigation of TME biology at the molecular and preclinical levels.

## References

[B1] Goenka A. Khan F. Verma B. Sinha P. Dmello C. Jogalekar M. P. (2023). Tumor microenvironment signaling and therapeutics in cancer progression. Cancer Commun. 43, 525–561. 10.1002/cac2.12416 37005490 PMC10174093

[B2] Tiwari A. Trivedi R. Lin S.-Y. (2022). Tumor microenvironment: barrier or opportunity towards effective cancer therapy. J. Biomed. Sci. 29, 83. 10.1186/s12929-022-00866-3 36253762 PMC9575280

[B3] Tonello S. Rolla R. Tillio P. Sainaghi P. Colangelo D. (2025). Microenvironment and tumor heterogeneity as pharmacological targets in precision oncology. Pharmaceuticals 18 (6), 915. 10.3390/ph18060915 40573308 PMC12195999

